# Accelerometry-Based Step Count Validation for Horse Movement Analysis During Stall Confinement

**DOI:** 10.3389/fvets.2021.681213

**Published:** 2021-06-22

**Authors:** Samantha L. Steinke, Julia B. Montgomery, John M. Barden

**Affiliations:** ^1^Biomedical Engineering, College of Engineering, University of Saskatchewan, Saskatoon, SK, Canada; ^2^Large Animal Clinical Sciences, Western College of Veterinary Medicine, University of Saskatchewan, Saskatoon, SK, Canada; ^3^Faculty of Kinesiology and Health Studies, University of Regina, Regina, SK, Canada

**Keywords:** motion tracking, equine, biomechanics, rehabilitation, inertial measurement unit, apple watch, accelerometer

## Abstract

Quantitative tracking of equine movement during stall confinement has the potential to detect subtle changes in mobility due to injury. These changes may warn of potential complications, providing vital information to direct rehabilitation protocols. Inertial measurement units (IMUs) are readily available and easily attached to a limb or surcingle to objectively record step count in horses. The objectives of this study were: (1) to compare IMU-based step counts to a visually-based criterion measure (video) for three different types of movements in a stall environment, and (2) to compare three different sensor positions to determine the ideal location on the horse to assess movement. An IMU was attached at the withers, right forelimb and hindlimb of six horses to assess free-movement, circles, and figure-eights recorded in 5 min intervals and to determine the best location, through analysis of all three axes of the triaxial accelerometer, for step count during stall confinement. Mean step count difference, absolute error (%) and intraclass correlation coefficients (ICCs) were determined to assess the sensor's ability to track steps compared to the criterion measure. When comparing sensor location for all movement conditions, the right-forelimb vertical-axis produced the best results (ICC = 1.0, % error = 6.8, mean step count difference = 1.3) followed closely by the right-hindlimb (ICC = 0.999, % error = 15.2, mean step count difference = 1.8). Limitations included the small number of horse participants and the lack of random selection due to limited availability and accessibility. Overall, the findings demonstrate excellent levels of agreement between the IMU's vertical axis and the video-based criterion at the forelimb and hindlimb locations for all movement conditions.

## Introduction

During recovery from an injury, horses are frequently confined to a stall for the initial phase of their rehabilitation. This confinement can result in restricted movement due to limited space and avoidance of painful weight-bearing on the injured limb. Movement is essential and aids the healing process by promoting circulation and the subsequent delivery of nutrients and oxygen to damaged tissues. Identification of reduced movements (e.g., walking) during stall confinement could significantly influence treatment outcomes by providing early recognition of complications and the implementation of preventative measures (1).

Although experienced veterinarians can subjectively assess most gait events (e.g., lameness), they are limited by the temporal resolution of the human eye and inter-observer agreement (2–5) and have been shown to disagree on the degree of lameness when observing the same horse at the same time (2–4, 6). Objective analysis removes these biases and decreases the need for a human to consistently monitor an animal. Small wearable sensors have the potential to provide accurate assessments of the type, speed, and quantity of movement (7, 8). Several studies have used inertial measurement units (IMUs) to quantify equine gait while walking or trotting in a straight line, but not during stall confinement (3, 7, 9–12) as it presents several potential challenges. Many different movements can occur (e.g., pawing and turning on the haunches or forehand), and movement is rarely at a consistent speed in a straight line. Although IMUs and accelerometers have been used to analyze equine movement, to our knowledge, no studies have tested IMUs during stall confinement. Other studies have looked at locomotor activity levels (11, 12), step frequency (11), footfall timing (6–8), stride rate, as well as timing and asymmetry (13) when walking in a straight line, on a lunge line or grazing in a paddock. Several of these studies referred to the best location for placement of the sensor. However, they did not include a direct comparison of the best anatomical location for a particular task and the sensor's validity for that task.

Therefore, the purpose of this study was to determine the concurrent validity of an IMU for equine step count determination during stall confinement compared to a visually-based (video) criterion. The specific objectives were: (1) to compare IMU and video-based step counts for three different movement types, and (2) to compare three different body positions and axes to determine the ideal location for IMU placement. It was hypothesized that the IMU would be capable of producing excellent levels of agreement (i.e., ICC > 0.75) at one or more of the test locations when compared to the video-based criterion measure.

## Materials and Methods

The study was approved by the University of Saskatchewan's Animal Research Ethics Board and adhered to the Canadian Council on Animal Care guidelines for humane animal use. For client-owned horses, informed client consent was obtained.

### Horses

Six horses of varying breed (Quarter Horses, Arabian/Paint cross, Thoroughbred; Mares and Geldings), sex and age (4–23 years old) participated in the study (Table 1). The number of horses was consistent with previous studies. However, there was a higher representation of middle-aged to older horses. None of the horses had been recently diagnosed with a musculoskeletal problem (11, 12). Two stalls of similar size were used in two different environments: a hospital stall (used to test two horses) and a stall within a personal barn (used to test four horses). The hospital stall was 16 ft (192 inches) by 14 ft (168 inches), and the personal barn was ~14.2 ft (170 inches) by 9.6 ft (115 inches). The testing took place in two different environments in an attempt to minimize the effect of several factors that could not be controlled in the original environment (a busy equine clinic). The horses used in the hospital setting had limited exposure to stall confinement, which increased the quantity of movement displayed in these trials due to pacing or stall walking. Additionally, the stall tests in the personal barn consisted of horses comfortable in a barn environment around familiar horses and handlers, which resulted in less movement for one of the conditions (free movement). The same handler completed all trials regardless of participant or location.

**Table 1:** The breed, age, and sex of each horse included in the study.

**Breed**	**Age (years)**	**Sex**
Quarter horse	18	Mare
Thoroughbred	23	Mare
Quarter horse	10	Gelding
Quarter horse	4	Gelding
Appendix quarter horse	20	Gelding
Arabian X paint	18	Gelding

### Movement Analysis

The protocol for determining IMU step count validity involved three different movement conditions: free movement, circles, and figure-eights. These movements were selected to mimic the different kinds of movements horses might display in a stall environment. The free movement condition simulated a horse in its natural environment, randomly moving around the stall for a specific period of time (5 min). Circles were selected to mimic a horse pacing around the stall, which occurred in some earlier preliminary trials. All six horses performed the free movement and circle conditions. The two horses tested in the hospital stall also completed a figure-eight movement pattern, as this would occur during stall confinement when attached to a computerized rehabilitation support device previously reported by our group (14). This movement was tested to ensure that the IMU could be used for movement monitoring in the figure-eight pattern during potential rehabilitative support situations.

Each horse completed four test sessions on separate days (see Supplementary Figure 1). Each session consisted of three 5 min free movement trials, one 5 min circle trial, and, if applicable, one 5 min figure-eight trial, completed consecutively. The circle and figure-eight movement conditions involved walking the horse around the stall in a circle or figure-eight pattern, respectively, for 5 min, reversing direction approximately half-way through. Three free movement trials were completed in each session (i.e., testing day) due to some trials having little to no movement. Trials with no movement were not included in the study.

### Equipment

**Figure 1:**
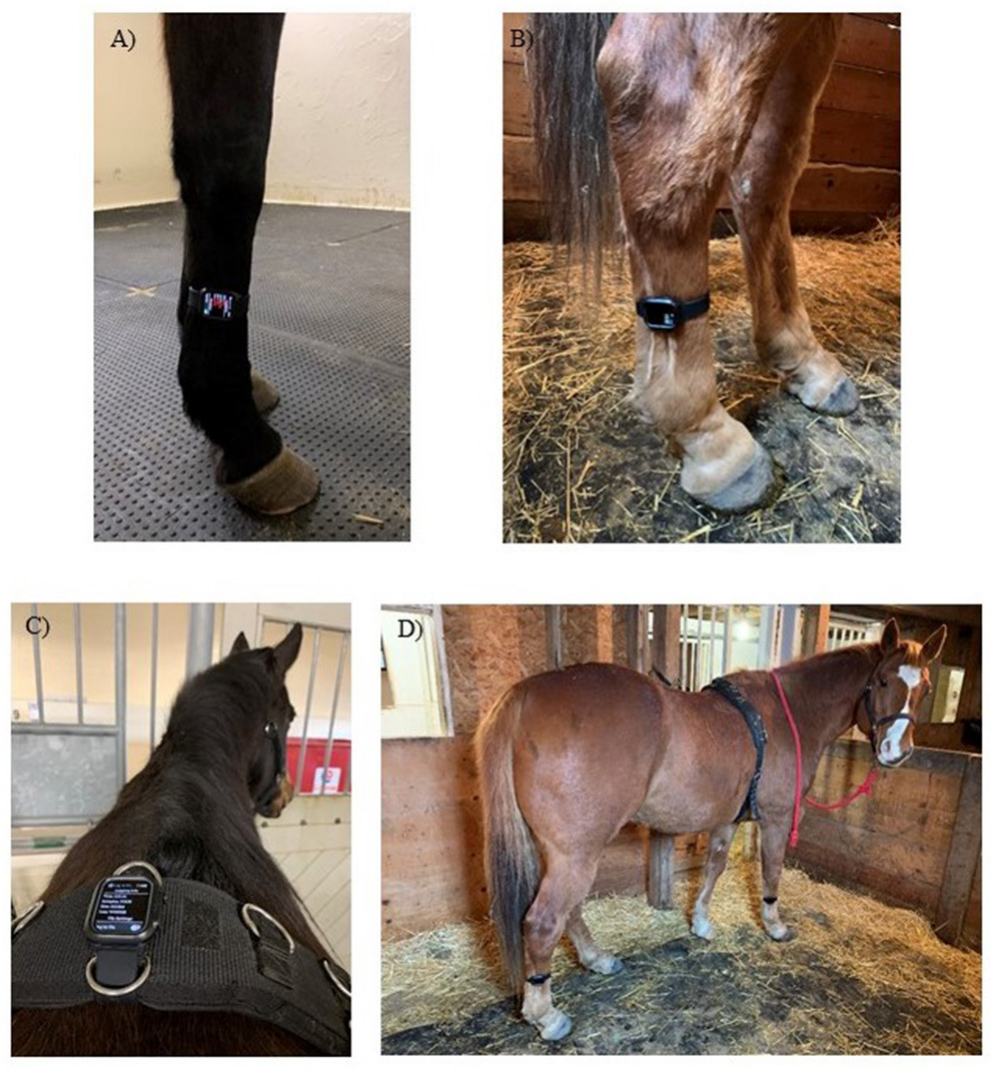
The locations in which the IMU was placed for movement analysis trials. **(A)** right forelimb cannon bone (metacarpus), **(B)** right hindlimb cannon bone (metatarsus), **(C)** withers attached to a surcingle and **(D)** IMUs at all three locations.

**Figure 2:**
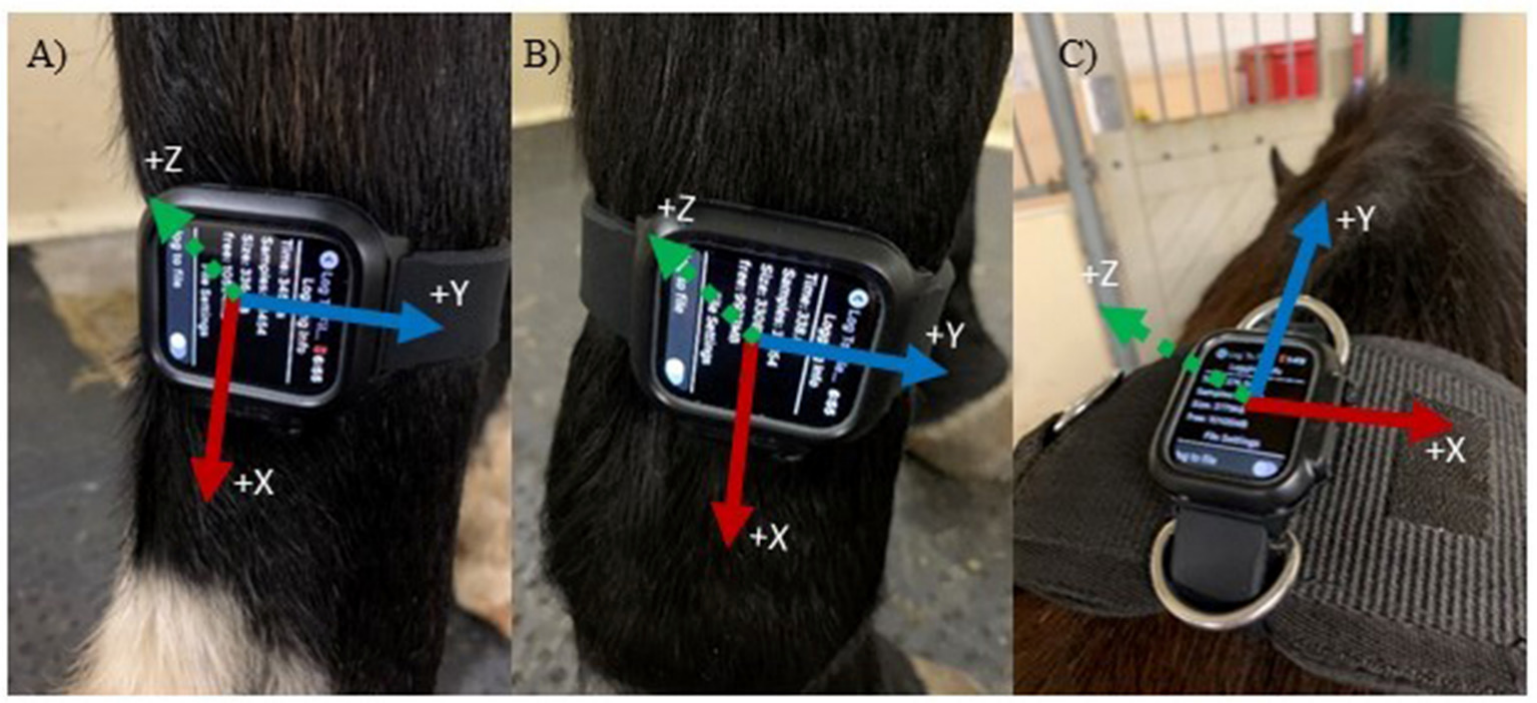
Accelerometer axes based on sensor location and orientation. Axes are indicated in the positive directions. **(A)** Right Hindlimb (Y = anteroposterior, X = vertical and Z = mediolateral); **(B)** Right Frontlimb (same axes as A); **(C)** Withers (Y = anteroposterior, X = mediolateral and Z = vertical).

Three IMUs (Apple Watch, Series 4, 44 mm, Apple Inc., Cupertino, CA, USA) with a range of ±32 g were placed on the right forelimb, right hindlimb and at the withers (Figure 1) to determine the best location for step count accuracy using data from all three axes of the IMU's triaxial accelerometer (Figure 2).

Three IMUs were used to permit data collection at all three locations simultaneously so that direct comparisons of different locations could be made for the same trial. The accelerometer-based step count was compared to the visually-determined step count to assess the sensor's validity for stall confinement movement analysis. A single observer analyzed the video recordings to determine the criterion step counts. All trials were recorded using a GoPro Hero 7 White (GoPro, Inc., San Mateo, CA, USA) video camera placed in the corner of the stall on the floor for clear visualization of the participants' limbs. The camera recorded at 60 frames per second (1,080 p resolution and a wide field of view) while the accelerometer sampled at 100 Hz. After the video recording was started, the IMUs were activated, 5 min of walking was recorded, and the video was stopped once all IMUs were deactivated. The 5 min trial period was determined based on the time elapsed on the GoPro video recording application, which was observed externally through a mobile phone.

The sensor locations were selected based on previous studies that used IMUs or accelerometers (10, 11, 15), as well as commercially available systems that contain accelerometers and gyroscopes (for example, the Lameness Locator) (10, 13, 16). The IMU's were attached to the right forelimb cannon bone (metacarpus), right hindlimb cannon bone (metatarsus) and the withers attached to a surcingle, which is consistent with other studies. Orientation was based on ease of use, ease of attachment and stability at that location. As a result, the limb IMUs were placed with a different orientation than the withers (Figures 1A,B vs. C). Only the recorded data from the IMU's accelerometer was used to determine step count, and coordination with the video was achieved via a hand signal in the video (thumbs up when activated and thumbs down when deactivated). The number of steps was determined between these two points. The IMU and video recordings were stopped and started between each trial to separate trials and movement conditions. Two free movement trials were removed from the final analysis due to no movement (steps) occurring in the video.

### Data Analysis

**Figure 3:**
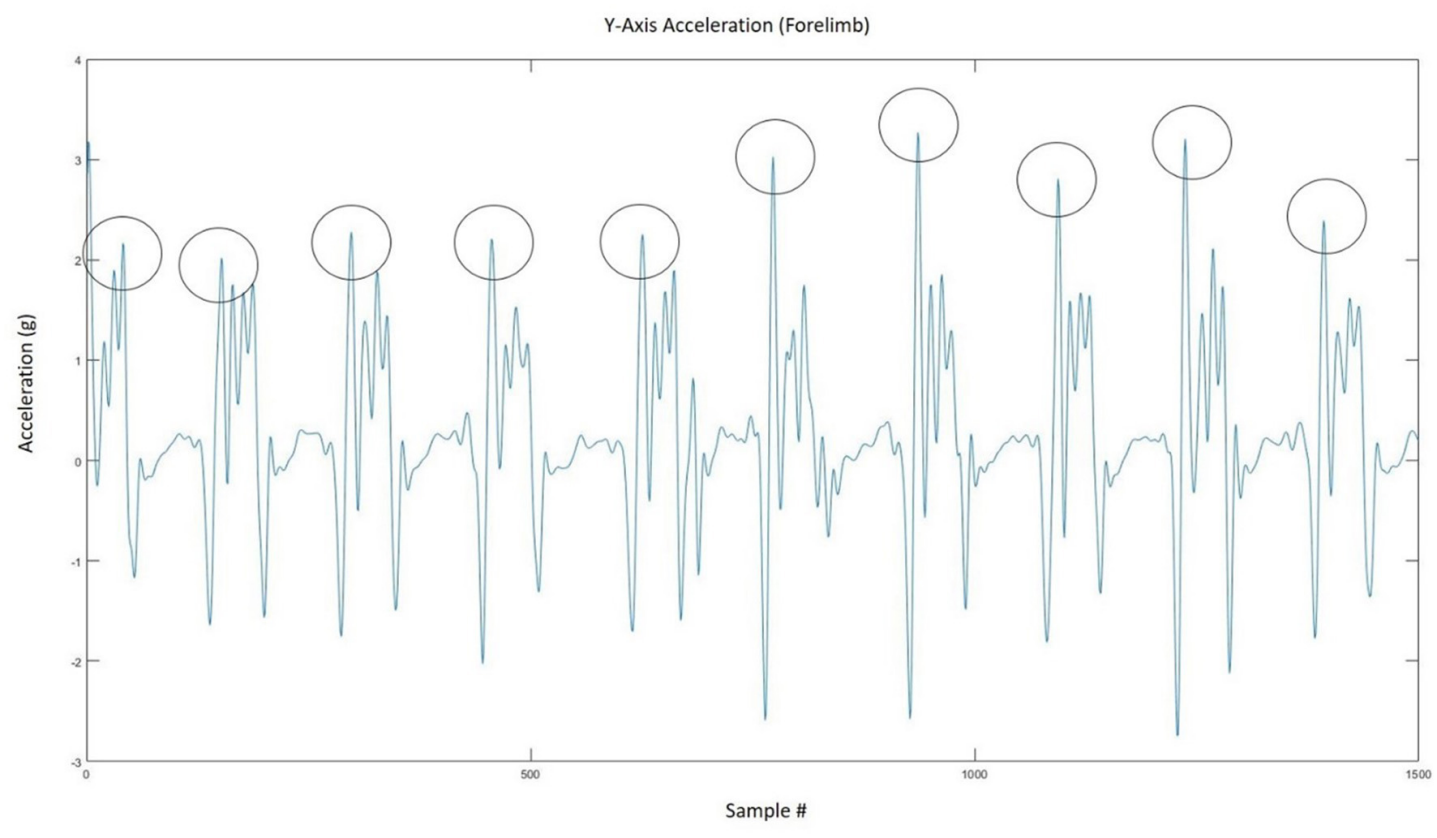
Representative figure showing how the peak detection algorithm extracted the number of steps from the accelerometer signal.

Accelerometer vs. video-based step counts were compared to determine sensor validity and the most accurate location for placement. A step was defined as the visual determination of lifting the heel off the ground and a flexed carpus or tarsus. When the accelerometer was placed on a limb, only the steps from that limb were counted, given that the accelerometer would only pick up movement from the limb to which it was attached. When the accelerometer was placed at the withers, the steps of both front legs were counted. The raw accelerometer data were processed using a zero-lag, fourth-order Butterworth low-pass filter set to a cut-off frequency of 10 Hz. A peak-detection algorithm (see Figure 3 for a segment of representative data and Appendix A for algorithms) that prescribed minimum time and amplitude-based thresholds for hoof-contact identification was implemented in MATLAB R2020a (The MathWorks Inc., Natick, MA, USA) to determine the series of step times for each axis (17, 18). Some devices (e.g., the Lameness Locator) only use uniaxial accelerometers (13). However, given the current proliferation and ubiquity of tri-axial accelerometers and the lack of equivalent movement in all three directions when walking, it was decided to investigate all three axes to determine the best axis for step count determination. The algorithm thresholds were refined through straight-line walking and walking in large circles in an arena to ensure consistent forward movement. Potential outliers were removed using a median filter that eliminated step times that were more or less than three standard deviations from the median (17, 18). This was done to minimize peak threshold detection errors (misidentifications and omissions) and to provide a more accurate step count. Step times that were too long or too short to be actual steps were replaced with the median step time, and the final series of step times was used to determine the step count for each trial. The algorithm used in this study was adapted from a similar algorithm that has been used to successfully detect steps and strides in humans (17, 18).

### Statistical Analysis

An intraclass correlation (ICC) was used to determine the absolute levels of agreement between the two methods (sensor step count vs. video-based step count). A model 3 (1, 3) ICC was used (19), which uses two-way mixed single measures to evaluate the agreement between methods on the dependent variable. Based on the ICCs, the level of agreement was classified as either poor (<0.4), moderate (0.4–0.75) or excellent (>0.75) (20). In addition to the ICC analysis, the absolute step count difference (number of steps) and absolute percent error (%) between methods for each trial was also determined. The absolute value of the percent error was determined using the following formula:

$$\begin{array}{l} Percent\;error=\frac{\left|video\;step\;count-sensor\;step\;count\right|}{\left(video\;step\;count\right)}*100 \end{array}$$

**Figure 4:**
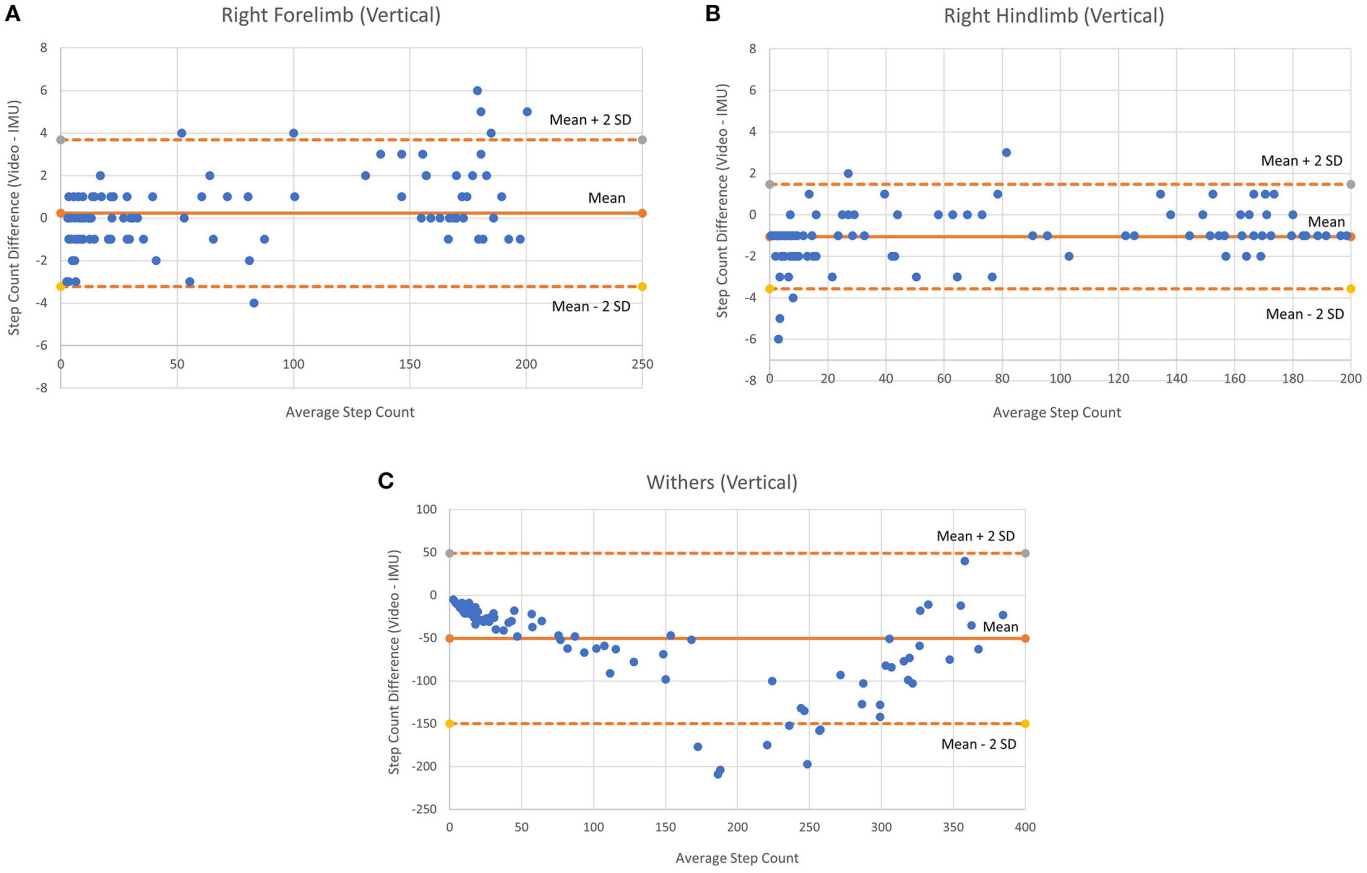
Bland-Altman plots for vertical axes of each sensor location (all movements combined). **(A)** Right Forelimb, **(B)** Right hindlimb, and **(C)** Withers.

Bland-Altman plots were also determined for each sensor location to visualize the limits of agreement between the two methods (Figure 4). The ICC analysis was conducted using IBM SPSS Statistics 26 (IBM, Armonk, NY, USA).

## Results

The ICC, mean % error and step count differences (for all movement trials combined) are shown in (Table 2). These calculations included 104 trials consisting of 14,534 steps at the withers location and 7,263 and 6,841 at the right forelimb and hindlimb locations, respectively. The vertical-axis at the right forelimb location had the highest level of agreement (1.00), lowest percent error (6.8%) and step count difference (1.3) when all movement conditions were combined (Table 2). The mediolateral axis was similar in ICC, percent error, and step count difference (0.999, 9.0% and 2.2, respectively). The ratings determined by the ICC were “excellent” in all trials, indicating a high level of agreement for the IMU sensor at all axes and locations.

**Table 2:** Results for the three accelerometer axes according to sensor location (all movements were combined to determine the best sensor location).

**Location**	**Axis**	**ICC**	**% error (Mean, SD)**	**Step count—video (Mean, SD)**	**Step count—IMU (Mean, SD)**	**Step count difference (Mean, SD)**
Withers	VL	0.923	60.3 (28.8)	139.8 (142.2)	89.3 (114.2)	51.2 (49.9)
	AP	0.974	43.6 (28.0)		111.1 (130.2)	31.3 (31.2)
	ML	0.992	20.7 (22.0)		144.5 (154.6)	15.4 (20.8)
Right Frontlimb	**VL**	**1.00**	**6.8 (11.7)**	69.8 (70.7)	**70.1 (71.5)**	**1.3 (1.2)**
	AP	0.997	21.3 (19.1)		76.1 (72.0)	6.3 (5.2)
	ML	0.999	9.0 (13.2)		71.1 (71.8)	2.2 (2.7)
Right Hindlimb	VL	0.999	15.2 (22.6)	65.8 (69.3)	64.3 (69.9)	1.8 (3.4)
	AP	0.988	23.4 (19.3)		76.1 (79.3)	11.1 (12.0)
	ML	0.990	21.7 (24.1)		72.3 (79.3)	8.7 (12.1)

*ML, Mediolateral; AP, Anteroposterior; VL, Vertical; SD, Standard Deviation. The right forelimb location had the highest level of agreement (ICC), lowest percent error and step count difference, which has been indicated in bold*.

Table 3 shows the ICC, mean % error and step count differences according to movement condition and sensor location. The free movement results included 71 trials (total steps: withers = 3,452; right forelimb = 1,732; right hindlimb = 1,531), the figure-eight results included 9 trials (total steps: withers = 3,010; right forelimb = 1,502; right hindlimb = 1,454) and the circle condition included 24 trials (total steps: withers = 7,895; right forelimb = 4,029; right hindlimb = 4,033). The vertical axis for the right forelimb produced the best results for the free movement condition (ICC = 0.999, % error = 9.6), while the vertical axis for the right hindlimb produced the best results for the figure-eight (ICC = 1.0; % error = 0.6) and circle conditions (ICC = 0.997; % error = 0.9). Figure 4 shows the Bland-Altman plots for each sensor location (all movement trials combined). In general, the plots show similar levels of agreement between the (Figures 4A,B) as the difference between methods was negligible (~0), but substantially more variation at the (Figure 4C) location, as the points were scattered further from the mean (a difference of −50 steps), especially for the larger step counts. Figure 4 also shows that the 95% confidence limits for the (Figure 4B) were slightly better than those for the (Figure 4A).

**Table 3:** Results for the three accelerometer axes according to movement condition and sensor location.

**Movement**	**Location**	**Axis**	**ICC**	**% error (Mean, SD)**	**Step count—video (Mean, SD)**	**Step count—IMU (Mean, SD)**	**Step count difference (Mean, SD)**
FM	W	VL	0.851	73.5 (22.2)	48.6 (49.4)	21.4 (32.7)	27.2 (19.2)
		AP	0.959	54.9 (25.9)		31.5 (45.2)	17.1 (9.1)
		ML	0.992	25.8 (24.4)		47.4 (55.0)	7.3 (6.0)
	RF	**VL**	**0.999**	**9.6 (13.3)**	24.4 (23.9)	**24.1 (24.1)**	**1.1 (1.0)**
		AP	0.976	29.3 (18.1)		30.5 (28.7)	6.1 (5.6)
		ML	0.997	12.2 (14.8)		25.0 (25.9)	1.9 (2.1)
	RH	VL	0.990	22.0 (24.6)	21.6 (23.4)	19.6 (23.6)	2.1 (4.1)
		AP	0.982	26.8 (21.9)		25.2 (26.8)	4.8 (4.7)
		ML	0.990	25.4 (27.8)		21.6 (24.8)	2.8 (3.9)
C	W	VL	0.505	30.1 (21.5)	329.0 (50.1)	241.7 (93.8)	93.7 (61.4)
		AP	0.704	19.3 (13.1)		281.6 (81.4)	60.8 (38.8)
		ML	0.853	10.4 (9.1)		352.4 (76.0)	35.9 (31.7)
	RF	VL	0.996	1.0 (1.0)	167.9 (18.4)	169.3 (18.2)	1.6 (1.7)
		AP	0.951	4.0 (3.2)		174.1 (16.1)	6.3 (4.7)
		ML	0.980	2.1 (2.1)		170.9 (16.3)	3.2 (3.8)
	RH	**VL**	**0.997**	**0.9 (1.3)**	168.0 (45.2)	**166.5 (41.4)**	**1.9 (4.5)**
		AP	0.831	17.4 (8.8)		190.7 (29.9)	28.1 (14.6)
		ML	0.270	17.1 (13.0)		182.3 (25.1)	30.7 (38.3)
F8	W	VL	0.429	33.4 (11.1)	334.4 (52.7)	225.2 (56.4)	109.2 (33.2)
		AP	0.683	18.8 (14.0)		274.4 (71.1)	60.0 (44.9)
		ML	0.928	6.6 (6.5)		339.7 (65.9)	22.8 (22.0)
	RF	VL	0.999	0.9 (0.7)	166.9 (26.1)	168 (26.5)	1.6 (1.3)
		AP	0.979	4.6 (1.9)		174.2 (25.9)	7.3 (2.3)
		ML	0.995	1.4 (3.2)		167.9 (22.8)	1.7 (3.2)
	RH	**VL**	**1.00**	**0.6 (0.4)**	161.6 (25.9)	**160.9 (26.1)**	**0.9 (0.6)**
		AP	0.857	12.6 (5.6)		181.7 (29.6)	20.1 (9.5)
		ML	0.907	9.1 (6.3)		175.6 (26.7)	14.0 (8.9)

*FM, Free Movement; C, Circle; F8, Figure-eight; W, Withers; RF, Right Forelimb; RH, Right Hindlimb; ML, Mediolateral; AP, Anteroposterior; VL, Vertical; SD, Standard Deviation. The right forelimb location had the highest level of agreement (ICC), lowest percent error and step count difference for free movement. The right hindlimb location had the highest level of agreement (ICC), lowest percent error and step count difference for circles and figure-eights. These values have been indicated in bold*.

## Discussion

This study investigated the concurrent validity of an IMU to determine equine step count during stall confinement. The hypothesis that the IMU-based method would achieve excellent levels of agreement (i.e., ICC > 0.75) compared to the video-based criterion was supported. The majority of axes and locations for all movement conditions had excellent levels of agreement (ICC > 0.75); however, several results did not meet this criterion, particularly for the anteroposterior and vertical axes at the withers location. Overall, IMU step counts agreed well with the criterion measure with more than half of the ICCs above 0.9, with low percent errors and step count differences. Consequently, the results demonstrate that IMUs are capable of accurately determining step count in horses during stall confinement.

Specifically, the vertical axis at the forelimb and hindlimb locations was the most accurate, and while both locations were best for different movements (e.g., free movement vs. figure-eight or circles), they behaved similarly in terms of outcome. Most importantly, the right forelimb had the highest accuracy for the combined movement analysis and in the free movement condition, which would be the most representative of a standard rehabilitation setting. When interpreting these results, it is important to appreciate that the ICC quantifies the level of agreement between two independently determined variables, in this case, the sensor-based step count and the video-based step count. Consequently, the ICC assesses the degree of similarity between these two measures. A high level of agreement will be achieved when a greater number of steps are counted in the video, and a corresponding greater number of steps are recorded by the sensor (and similarly for a lower number of steps). However, the absolute percent error (based on the absolute step count difference) measures the proportional discrepancy between the two measures. In addition to the level of agreement, this discrepancy is important because, in a rehabilitation context, a comparison of values across hours or days would be used to determine how much a movement had increased or decreased. In the presence of large step count errors, between-day comparisons may not have sufficient accuracy to identify small changes in movement. Accuracy is key in being able to detect changes early and possibly prevent life-threatening complications. Unfortunately, acceptable levels of agreement and absolute errors for equine rehabilitation monitoring have yet to be determined. Future studies on injured horses should attempt to determine clinically significant levels of movement reduction associated with conditions such as muscle wasting, osteopenia, or supporting limb laminitis (SLL). It is also not known how long movement reductions can persist before veterinarians need to intervene. If an IMU is used to monitor recovery from injury, daily step counts that indicate slight declines over time, could suggest that more significant interventions are needed to increase activity to promote cyclic loading and blood flow, preventing life-threatening complications from developing.

The results of this study are comparable to those reported by Fries et al. (11), who used four locations (withers, forelimb, hindlimb, and head) to evaluate whether an accelerometer could quantify locomotor activity and step count in horses while grazing (5 min.), walking at different speeds, trotting, and cantering. A free movement analysis was also conducted for 20 min inside a paddock, which was likely larger than a stall. The findings were similar to this study in that the hindlimb location was found to be most accurate (forelimb and hindlimb in our study), while the withers location was problematic. They concluded that percent errors and limits of agreement were not acceptable for step count at the withers, head, and forelimb. In contrast, this study found lower percent errors and excellent levels of agreement at the forelimb location. This discrepancy may have occurred as the result of a difference in sensor placement, given that Fries et al. (11) placed the accelerometer at the heel as opposed to the cannon bone. Consequently, the current results suggest that the sensor should be placed on the cannon bone, as other studies have also achieved good results at this location (7).

The most important finding of this study pertains to the free movement condition, given that rehabilitation monitoring would likely consist of monitoring a horse in a stall with random intermittent movements. Injured horses that require monitoring are less likely to pace around the stall if they are in pain. As such, the reason for monitoring a horse is usually insufficient movement, which may be indicative of inadequate pain control or the development of immobility-associated complications. Measuring movement in injured horses could prove to be challenging with added alterations in movement, such as reduced and altered loading patterns due to pain, changes in gait patterns due to asymmetries (2, 5, 21–23), as well as decreased stability and increased postural sway (24), all of which can be affected to varying degrees depending on the type, location and severity of the injury (25). Consequently, free movement analysis would likely be the most important measure in these circumstances. Due to the diverse and unpredictable nature of free movements, it is considerably more difficult to fine-tune an algorithm to discriminate between steps and other behaviors, as opposed to consistent forward movements, in which gait parameters such as step time and step length are relatively constant. The high ICC (0.999), low percent error (9.6%) and step count difference (1.1) in the free movement condition demonstrates that the algorithm was able to measure steps even with considerable variation in the types of movements performed during stall confinement. This movement variability is shown by the larger standard deviation and decreased accuracy during free movements compared to the circles and figure-eight conditions. There was also greater variability between horses, as some horses moved around the stall more frequently than others. The difficulty in tracking such movement lies in inter-individual variability, not only in individual differences in movement but also the amount of movement, which is also shown in the Bland-Altman plots. The limits of agreement were slightly better for the hindlimb location. However, interestingly, there was a slight bias (~−1.0) for the sensor to under-estimate steps at this location, which was not present at the forelimb location. Nevertheless, the results show great promise in being able to detect a clinically significant difference in the number of steps between a healthy horse and an injured horse, as this difference would likely be greater than the confidence limits shown. In the future, other parameters such as gait speed (8) and foot contact/lift-off (7) could be used in combination with step count to analyze movement within a stall, and the level of detection accuracy could be improved with the addition of machine-learning algorithms that can be trained to accurately detect movements with greater variability and at slower speeds.

Finally, it should be noted that this study has several limitations, including potential errors in the criterion step count determination (e.g., a single examiner was used to determine all video-based step counts) and misidentification errors in the raw accelerometer data by the peak-detection thresholds. It was also not possible to randomly select the participants, as the horses that did participate did so based on accessibility and availability. This resulted in a higher proportion of older and middle-aged horses in the sample, which, while not affecting the comparison between the methods, could have affected the frequency and speed of movements as well as the total number of steps. In addition, the horses may have moved less if they were uncomfortable with the sensation or the placement of the IMU's, but this is unlikely to have affected the results given the total number of trials, movements, locations and steps that were analyzed. Future studies should compare IMU and video-based methods using a larger sample of horses and additional gait and/or stall-related movement parameters, such as duration of time spent walking vs. standing, limb kinematics and postural sway when standing for prolonged periods of time. The results of this study suggest that the development of an iOS or Android app to track movement and provide updates over days or weeks has the potential to provide a substantial benefit to the practice of equine rehabilitation.

## Conclusion

This study found that the vertical axis of a sensor placed at the forelimb or hindlimb was the most accurate to determine step count in a stall environment. Excellent levels of agreement suggest that an IMU could continuously monitor the quantity of movement in a stall, which would indicate any abnormalities associated with reduced movement before those changes could become severe. The results show that accelerometry has the potential to provide an easy and valuable tool for veterinary medicine in the early detection of movement reduction that could signal increasing levels of pain or immobility-associated complications. This early detection could provide an opportunity for timely preventative interventions during the injury recovery and rehabilitation process, possibly leading to more successful outcomes.

## Data Availability Statement

The raw data supporting the conclusions of this article will be made available by the authors, without undue reservation.

## Ethics Statement

The animal study was reviewed and approved by the University of Saskatchewan's Animal Research Ethics Board and adhered to the Canadian Council on Animal Care guidelines for humane animal use. Written informed consent was obtained from the owners for the participation of their animals in this study.

## Author Contributions

SS wrote the article. SS was a graduate student in biomedical engineering co-supervised by JM and JB. The field of research is equine biomechanics and rehabilitation. All authors revised the manuscript and approved the final version for submission.

## Conflict of Interest

The authors declare that the research was conducted in the absence of any commercial or financial relationships that could be construed as a potential conflict of interest.
