# Supplementary material for: Accelerometry-Based Step Count Validation for Horse Movement Analysis During Stall Confinement
Source: Front Vet Sci. 2021 Jun 22;8:681213. doi: 10.3389/fvets.2021.681213 (PMC8259880; doi:10.3389/fvets.2021.681213)
Supplement: Supplementary file 1 [file Data_Sheet_1.pdf]

Supplementary Material

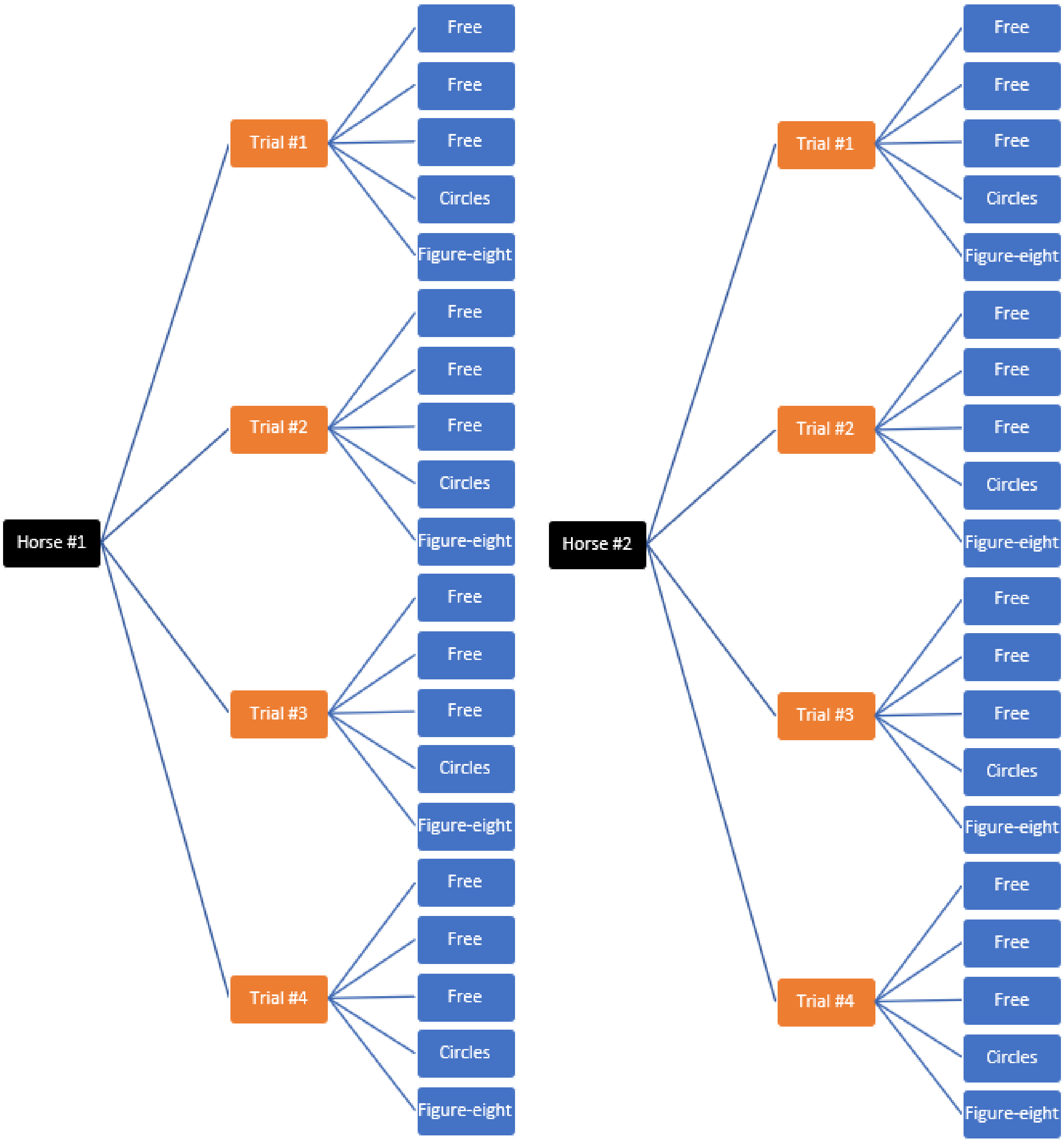

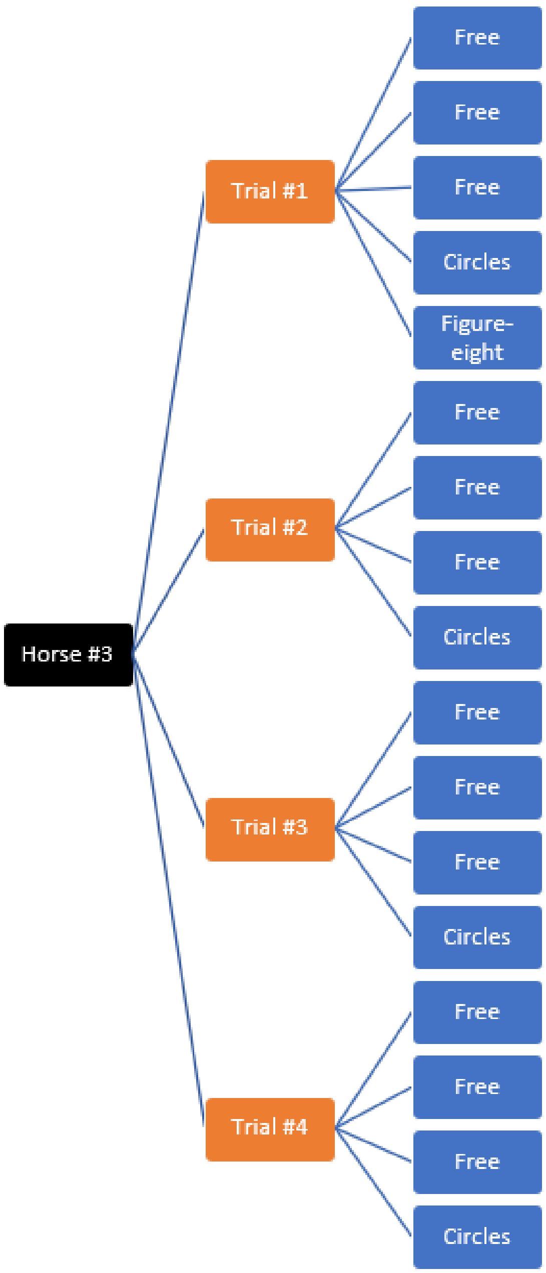
Horse #3
Trial #1
Free
Free
Free
Circles
Figure-eight
Trial #2
Free
Free
Free
Circles
Trial #3
Free
Free
Free
Circles
Trial #4
Free
Free
Free
Circles

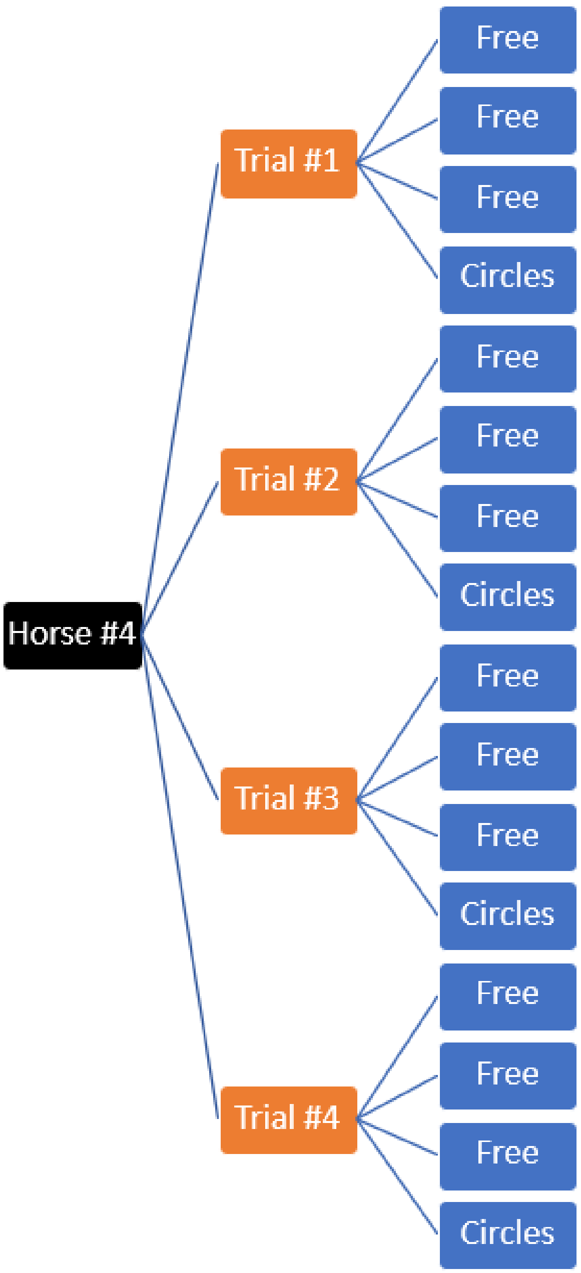
Horse #4
Trial #1
Free
Free
Free
Circles
Trial #2
Free
Free
Free
Circles
Trial #3
Free
Free
Free
Circles
Trial #4
Free
Free
Free
Circles

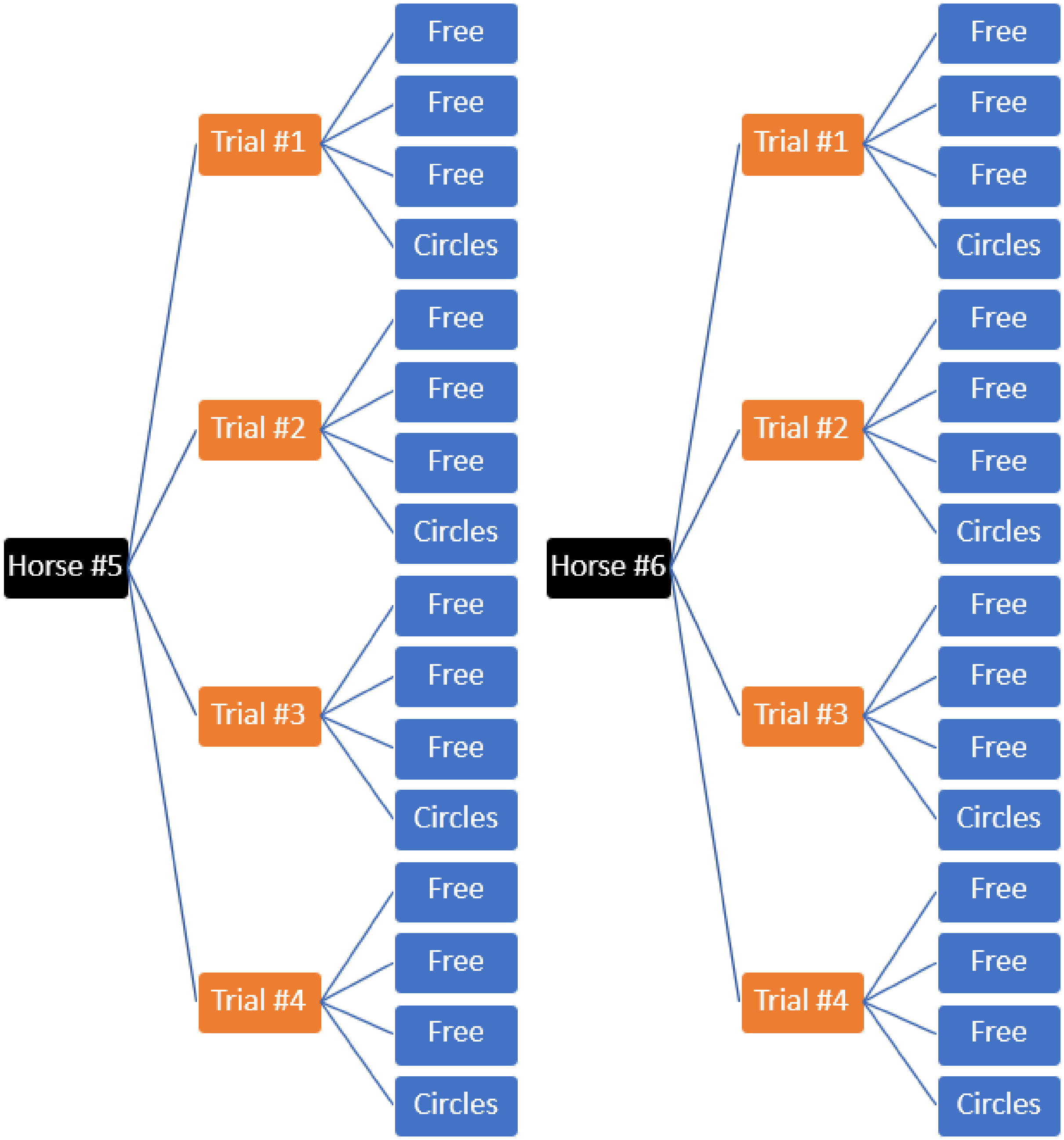

Figure S1. The layout of testing completed by each horse. Each horse completed four days of testing. Each test consisted of three five-minute free movements, one five-minute circle and, if applicable, one five-minute figure-eight. Three free movements were completed in each test due to some free movements having little to no movement. 24 testing sessions in total were completed, including 72 Free movement trials (3 per test day), 24 circle trials, and nine figure-eight trials (the smallest horse at the personal barn attempted the figure-eights in the first test session with difficulty, due to stall size. Therefore, it was removed from future sessions in this location).

1 APPENDIX A: MATLAB CODES

LPFilt100 CODE

```
%This script lowpass filters accelerometer data that has been
imported as
%follows.

    %Note re: placement and axes. The Apple Watch IMU should be
placed at the withers, right forelimb or hindlimb

    %Import data: To load the data from a .csv file, rename the
file 'Data', then block the 3 data columns to the right,
    %select 'Numeric Matrix' as the output type and then click
'Import data' under 'Import Selection'. Save the variable 'Data'
    % as a Matlab file ('Data.mat'). The data should be contained
in the data file Data.mat.

    %n = the order of the filter (default is 4) Fc = the low pass
cutoff
    %frequency (default is 10 Hz) Fs = the sampling rate (default
    %is 100 Hz) Wn = the normalized cutoff frequency (this should
be 0.2,
    %regardless of whether the sampling is 100 or 60 Hz (i.e., use
10 Hz
    %cutoff for 100 Hz sampling rate and 6 Hz for 60 Hz sampling;
Wn will =
    %0.2 in both cases). b and a = the filter coefficients
    load Data.mat
%Loads the imported data structure file.
    X = Data (2:end, 1); % Creates the variable X for the 1st
column (= the ML accel. data).
    Y = Data (2:end, 2); % Creates the variable Y for the 2nd
column (= the Vertical accel. data)
    Z = Data (2:end, 3); % Creates the variable Z for the 3rd
column (= the AP accel. data)

    n = 4;
    Fc = 10;
    Fs = 100;
    Wn = (Fc*2)/Fs;
    [b,a] = butter(n, Wn, 'low');
    filtx = filtfilt(b, a, X);
    filty = filtfilt(b, a, Y);
    filtz = filtfilt(b, a, Z);
```

STV_X CODE (Limbs)

```
  %This is the PREFERRED routine for processing the vertical
signal.

    %This script finds the time between the LP filtered X-axis
peaks (i.e, the individual Step Times) and removes outliers that
are
    %3 SD above and below the median Step time.

    %findpeaks is the function that finds the peaks of the signal.
    %pks = the magnitude of the peaks.
    %locs = the location (i.e, the sample number) of the peaks
(i.e., the
    %peak locations).

    [pks, locs] = findpeaks(filtx,'MinPeakDistance',80,
'MinPeakProminence', 0.5); %This command finds the peaks and
    %creates variables for the magnitude (pks) and locations (locs)
of the peaks. The command also specifies that
    %there must be a minimum horizontal distance between each peak
(i.e,. default = 20 samples; i.e., 0.33 s @ 60 Hz)
    %and that the peaks must be 0.30 g higher than the lowest
value.
    SteptimeX = diff(locs) * 1/Fs; %This command finds the
differences between the peak locations (i.e., # of samples)
    %and then multiplies this by the sampling rate time. This
provides the
    %series of individual step times.

    ThreshU = median(SteptimeX) + 3*(std(SteptimeX)); %This command
finds the median value of the SteptimeX variable
    %and then adds 3 standard deviations to it.
    OutliersU = find(SteptimeX > ThreshU); %This creates a variable
that contains the outliers that are greater than the
    %Threshold value.
    SteptimeX(OutliersU) = [median(SteptimeX)]; %This command
replaces the outliers in SteptimeX with the median
    %steptime.
    %The nextseries of commands repeats the above process for
steptimes
    %that are 3 SD's below the median step time.
    ThreshD = median(SteptimeX) - 3*(std(SteptimeX));
    OutliersD = find(SteptimeX < ThreshD);
    SteptimeX(OutliersD) = [median(SteptimeX)];

    Odd = SteptimeX(1:2:end,:);  %This creates a variable of odd
steptimes.
    Even = SteptimeX(2:2:end,:);  %This creates a variable of even
steptimes.
```

```
    SizeO = size (Odd,1); %This provides the number of rows in the
Odd steptime variable.
    SizeE = size (Even,1); %This provides the number of rows in the
Even steptime variable.

    %The "if elseif" statement below says: if the size (i.e., # of
rows) of
    %the Odd and Even variables are the same, then Odd = Odd (i.e.,
do
    %nothing). If the size of Odd is greater than Even (which will
occur
    %when you have an odd number of rows) then the last row in the
Odd variable is to be removed (Odd(SizeO(1),:) = [];).

    if SizeO == SizeE
        Odd = Odd;
    elseif SizeO > SizeE;
    Odd(SizeO(1),:) = [];
    end

    StridetimeX = Odd + Even;  %This statement adds each row of the
Odd and Even variables together to provide the series of
    %stride times.
    AsymX = (abs(mean(Odd) - mean(Even))/((mean(Odd) +
mean(Even))/2))*100; %This command finds the asymmetry between
    %the mean of the right and left steps.
    avgStepX = mean(SteptimeX); %This variable finds the mean of
the series of step times.
    avgStrideX = mean(StridetimeX); %This variable find the mean of
the series of stride times.
    COVStepX = (std(SteptimeX)/avgStepX) *100; %This variable finds
the coefficient of variation of the step time series.
    COVStrideX = (std(StridetimeX)/avgStrideX) *100; %This variable
finds the coefficient of variation of the stride time
    %series.
    [CadenceX] = 1/((size(filtx)/size(SteptimeX))/60)*60; %This
variable determines the overall cadence of the series
    %based on the total number of samples (in filtx) and the total
number of
    %steps (SteptimeX).
    StepcountX = SizeE + SizeO;
    save StepX.txt SteptimeX -ascii %This saves the variable
Steptime (containing the series of step times) to a .txt file.
    save StrideX.txt StridetimeX -ascii %This saves the variable
Stridetime (containing the series of stride times) to
    %a .txt file.
    %The following line saves the output variables to a ascii .txt
file
```

```
    %(figure out how to include a column of labels).
    save OutputX.txt StepcountX avgStepX COVStepX CadenceX AsymX -
ascii
```

STV_XW CODE (Withers)

```
%This is an alternative routine for processing the "withers"
mediolateral signal.

    %This script finds the time between the LP filtered X-axis
peaks (i.e, the individual Step Times) and removes outliers that
are
    %3 SD above and below the median Step time.

    %findpeaks is the function that finds the peaks of the signal.
    %pks = the magnitude of the peaks.
    %locs = the location (i.e, the sample number) of the peaks
(i.e., the
    %peak locations).

    [pks, locs] = findpeaks(filtx,'MinPeakDistance',35,
'MinPeakProminence', 0.3); %This command finds the peaks and
    %creates variables for the magnitude (pks) and locations (locs)
of the peaks. The command also specifies that
    %there must be a minimum horizontal distance between each peak
(i.e,. default = 20 samples; i.e., 0.33 s @ 60 Hz)
    %and that the peaks must be 0.30 g higher than the lowest
value.
    SteptimeX = diff(locs) * 1/Fs; %This command finds the
differences between the peak locations (i.e., # of samples)
    %and then multiplies this by the sampling rate time. This
provides the
    %series of individual step times.

    ThreshU = median(SteptimeX) + 3*(std(SteptimeX)); %This command
finds the median value of the SteptimeX variable
    %and then adds 3 standard deviations to it.
    OutliersU = find(SteptimeX > ThreshU); %This creates a variable
that contains the outliers that are greater than the
    %Threshold value.
    SteptimeX(OutliersU) = [median(SteptimeX)]; %This command
replaces the outliers in SteptimeX with the median
    %steptime.
    %The nextseries of commands repeats the above process for
steptimes
    %that are 3 SD's below the median step time.
    ThreshD = median(SteptimeX) - 3*(std(SteptimeX));
    OutliersD = find(SteptimeX < ThreshD);
    SteptimeX(OutliersD) = [median(SteptimeX)];
```

```
    Odd = SteptimeX(1:2:end,:);  %This creates a variable of odd
steptimes.
    Even = SteptimeX(2:2:end,:);  %This creates a variable of even
steptimes.
    SizeO = size (Odd,1); %This provides the number of rows in the
Odd steptime variable.
    SizeE = size (Even,1); %This provides the number of rows in the
Even steptime variable.

    %The "if elseif" statement below says: if the size (i.e., # of
rows) of
    %the Odd and Even variables are the same, then Odd = Odd (i.e.,
do
    %nothing). If the size of Odd is greater than Even (which will
occur
    %when you have an odd number of rows) then the last row in the
Odd variable is to be removed (Odd(SizeO(1),:) = [];).

    if SizeO == SizeE
        Odd = Odd;
    elseif SizeO > SizeE;
    Odd(SizeO(1),:) = [];
    end

    StridetimeX = Odd + Even;  %This statement adds each row of the
Odd and Even variables together to provide the series of
    %stride times.
    AsymX = (abs(mean(Odd) - mean(Even))/((mean(Odd) +
mean(Even))/2))*100; %This command finds the asymmetry between
    %the mean of the right and left steps.
    avgStepX = mean(SteptimeX); %This variable finds the mean of
the series of step times.
    avgStrideX = mean(StridetimeX); %This variable find the mean of
the series of stride times.
    COVStepX = (std(SteptimeX)/avgStepX) *100; %This variable finds
the coefficient of variation of the step time series.
    COVStrideX = (std(StridetimeX)/avgStrideX) *100; %This variable
finds the coefficient of variation of the stride time
    %series.
    [CadenceX] = 1/((size(filtx)/size(SteptimeX))/60)*60; %This
variable determines the overall cadence of the series
    %based on the total number of samples (in filty) and the total
number of
    %steps (SteptimeX).
    StepcountX = SizeE + SizeO;
    save StepX.txt SteptimeX -ascii %This saves the variable
Steptime (containing the series of step times) to a .txt file.
```

```
    save StrideX.txt StridetimeX -ascii %This saves the variable
Stridetime (containing the series of stride times) to
    %a .txt file.
    %The following line saves the output variables to a ascii .txt
file
    %(figure out how to include a column of labels).
    save OutputX.txt StepcountX avgStepX COVStepX CadenceX AsymX -
ascii
```

STV_Y CODE (Limbs)

```
%This is the PREFERRED routine for processing the AP signal.

    %This script finds the time between the LP filtered Y-axis
peaks (i.e, the individual Step Times) and removes outliers that
are
    %3 SD above and below the median Step time.

    %findpeaks is the function that finds the peaks of the signal.
    %pks = the magnitude of the peaks.
    %locs = the location (i.e, the sample number) of the peaks
(i.e., the
    %peak locations).

    [pks, locs] = findpeaks(filty,'MinPeakDistance',80,
'MinPeakProminence', 0.5); %This command finds the peaks and
    %creates variables for the magnitude (pks) and locations (locs)
of the peaks. The command also specifies that
    %there must be a minimum horizontal distance between each peak
(i.e,. default = 20 samples; i.e., 0.33 s @ 60 Hz)
    %and that the peaks must be 0.30 g higher than the lowest
value.
    SteptimeY = diff(locs) * 1/Fs; %This command finds the
differences between the peak locations (i.e., # of samples)
    %and then multiplies this by the sampling rate time. This
provides the
    %series of individual step times.

    ThreshU = median(SteptimeY) + 3*(std(SteptimeY)); %This command
finds the median value of the SteptimeY variable
    %and then adds 3 standard deviations to it.
    OutliersU = find(SteptimeY > ThreshU); %This creates a variable
that contains the outliers that are greater than the
    %Threshold value.
    SteptimeY(OutliersU) = [median(SteptimeY)]; %This command
replaces the outliers in SteptimeY with the median
    %steptime.
```

```
    %The nextseries of commands repeats the above process for
steptimes
    %that are 3 SD's below the median step time.
    ThreshD = median(SteptimeY) - 3*(std(SteptimeY));
    OutliersD = find(SteptimeY < ThreshD);
    SteptimeY(OutliersD) = [median(SteptimeY)];

    Odd = SteptimeY(1:2:end,:);  %This creates a variable of odd
steptimes.
    Even = SteptimeY(2:2:end,:);  %This creates a variable of even
steptimes.
    SizeO = size (Odd,1); %This provides the number of rows in the
Odd steptime variable.
    SizeE = size (Even,1); %This provides the number of rows in the
Even steptime variable.

    %The "if elseif" statement below says: if the size (i.e., # of
rows) of
    %the Odd and Even variables are the same, then Odd = Odd (i.e.,
do
    %nothing). If the size of Odd is greater than Even (which will
occur
    %when you have an odd number of rows) then the last row in the
Odd variable is to be removed (Odd(SizeO(1),:) = [];).

    if SizeO == SizeE
        Odd = Odd;
    elseif SizeO > SizeE;
    Odd(SizeO(1),:) = [];
    end

    StridetimeY = Odd + Even;  %This statement adds each row of the
Odd and Even variables together to provide the series of
    %stride times.
    AsymY = (abs(mean(Odd) - mean(Even))/((mean(Odd) +
mean(Even))/2))*100; %This command finds the asymmetry between
    %the mean of the right and left steps.
    avgStepY = mean(SteptimeY); %This variable finds the mean of
the series of step times.
    avgStrideY = mean(StridetimeY); %This variable find the mean of
the series of stride times.
    COVStepY = (std(SteptimeY)/avgStepY) *100; %This variable finds
the coefficient of variation of the step time series.
    COVStrideY = (std(StridetimeY)/avgStrideY) *100; %This variable
finds the coefficient of variation of the stride time
    %series.
    [CadenceY] = 1/((size(filty)/size(SteptimeY))/60)*60; %This
variable determines the overall cadence of the series
```

```
    %based on the total number of samples (in filty) and the total
number of
    %steps (SteptimeY).
    StepcountY = SizeE + SizeO;
    save StepY.txt SteptimeY -ascii %This saves the variable
Steptime (containing the series of step times) to a .txt file.
    save StrideY.txt StridetimeY -ascii %This saves the variable
Stridetime (containing the series of stride times) to
    %a .txt file.
    %The following line saves the output variables to a ascii .txt
file
    %(figure out how to include a column of labels).
    save OutputY.txt StepcountY avgStepY COVStepY CadenceY AsymY -
ascii
```

STV_YW CODE (Withers)

```
  %This is the PREFERRED routine for processing the AP signal.

    %This script finds the time between the LP filtered Y-axis
peaks (i.e, the individual Step Times) and removes outliers that
are
    %3 SD above and below the median Step time.

    %findpeaks is the function that finds the peaks of the signal.
    %pks = the magnitude of the peaks.
    %locs = the location (i.e, the sample number) of the peaks
(i.e., the
    %peak locations).

    [pks, locs] = findpeaks(filty,'MinPeakDistance',35,
'MinPeakProminence', 0.3); %This command finds the peaks and
    %creates variables for the magnitude (pks) and locations (locs)
of the peaks. The command also specifies that
    %there must be a minimum horizontal distance between each peak
(i.e,. default = 20 samples; i.e., 0.33 s @ 60 Hz)
    %and that the peaks must be 0.30 g higher than the lowest
value.
    SteptimeY = diff(locs) * 1/Fs; %This command finds the
differences between the peak locations (i.e., # of samples)
    %and then multiplies this by the sampling rate time. This
provides the
    %series of individual step times.

    ThreshU = median(SteptimeY) + 3*(std(SteptimeY)); %This command
finds the median value of the SteptimeY variable
    %and then adds 3 standard deviations to it.
    OutliersU = find(SteptimeY > ThreshU); %This creates a variable
that contains the outliers that are greater than the
```

```
    %Threshold value.
    SteptimeY(OutliersU) = [median(SteptimeY)]; %This command
replaces the outliers in SteptimeY with the median
    %steptime.
    %The nextseries of commands repeats the above process for
steptimes
    %that are 3 SD's below the median step time.
    ThreshD = median(SteptimeY) - 3*(std(SteptimeY));
    OutliersD = find(SteptimeY < ThreshD);
    SteptimeY(OutliersD) = [median(SteptimeY)];

    Odd = SteptimeY(1:2:end,:);  %This creates a variable of odd
steptimes.
    Even = SteptimeY(2:2:end,:);  %This creates a variable of even
steptimes.
    SizeO = size (Odd,1); %This provides the number of rows in the
Odd steptime variable.
    SizeE = size (Even,1); %This provides the number of rows in the
Even steptime variable.

    %The "if elseif" statement below says: if the size (i.e., # of
rows) of
    %the Odd and Even variables are the same, then Odd = Odd (i.e.,
do
    %nothing). If the size of Odd is greater than Even (which will
occur
    %when you have an odd number of rows) then the last row in the
Odd variable is to be removed (Odd(SizeO(1),:) = [];).

    if SizeO == SizeE
        Odd = Odd;
    elseif SizeO > SizeE;
    Odd(SizeO(1),:) = [];
    end

    StridetimeY = Odd + Even;  %This statement adds each row of the
Odd and Even variables together to provide the series of
    %stride times.
    AsymY = (abs(mean(Odd) - mean(Even))/((mean(Odd) +
mean(Even))/2))*100; %This command finds the asymmetry between
    %the mean of the right and left steps.
    avgStepY = mean(SteptimeY); %This variable finds the mean of
the series of step times.
    avgStrideY = mean(StridetimeY); %This variable find the mean of
the series of stride times.
    COVStepY = (std(SteptimeY)/avgStepY) *100; %This variable finds
the coefficient of variation of the step time series.
```

```
    COVStrideY = (std(StridetimeY)/avgStrideY) *100; %This variable
finds the coefficient of variation of the stride time
    %series.
    [CadenceY] = 1/((size(filty)/size(SteptimeY))/60)*60; %This
variable determines the overall cadence of the series
    %based on the total number of samples (in filty) and the total
number of
    %steps (SteptimeY).
    StepcountY = SizeE + SizeO;
    save StepY.txt SteptimeY -ascii %This saves the variable
Steptime (containing the series of step times) to a .txt file.
    save StrideY.txt StridetimeY -ascii %This saves the variable
Stridetime (containing the series of stride times) to
    %a .txt file.
    %The following line saves the output variables to a ascii .txt
file
    %(figure out how to include a column of labels).
    save OutputY.txt StepcountY avgStepY COVStepY CadenceY AsymY -
ascii
```

STV_Z CODE (Limbs)

```
  %This is the PREFERRED routine for processing the ML signal.

    %This script finds the time between the LP filtered Z-axis (AP)
peaks (i.e, the individual Step times) and removes outliers that
are
    %3 SD above and below the median Step time.

    %findpeaks is the function that finds the peaks of the signal.
    %pks = the magnitude of the peaks.
    %locs = the location (i.e, the sample number) of the peaks
(i.e., the
    %peak locations).

    Negz = filtz * -1; %This command flips the LP filtered Z-axis
signal (so that the peak minimums are now maximums).
    [pks, locs] = findpeaks(Negz,'MinPeakDistance',80,
'MinPeakProminence', 0.50); %This command finds the peaks and
creates variables
    %for the magnitude (pks) and locations (locs) of the peaks. The
command also specifies that there must
    %be a minimum horizontal distance between each peak (i.e,. 20
samples = 0.33 s)
    %and that the peaks must be 0.3 g higher than the lowest value.
    SteptimeZ = diff(locs) * 1/Fs; %This command finds the
differences between the peak locations (i.e., # of samples)
```

```
    %and then multiplies this by the sampling rate time. This
provides the
    %series of individual step times.

    ThreshU = median(SteptimeZ) + 3*(std(SteptimeZ)); %This command
finds the median value of the SteptimeZ variable
    %and then adds 3 standard deviations to it.
    OutliersU = find(SteptimeZ > ThreshU); %This creates a variable
that contains the outliers that are greater than the
    %Threshold value.
    SteptimeZ(OutliersU) = [median(SteptimeZ)]; %This command
replaces the outliers in SteptimeZ with the median
    %steptime.
    %The nextseries of commands repeats the above process for
steptimes
    %that are 3 SD's below the median step time.
    ThreshD = median(SteptimeZ) - 3*(std(SteptimeZ));
    OutliersD = find(SteptimeZ < ThreshD);
    SteptimeZ(OutliersD) = [median(SteptimeZ)];

    Odd = SteptimeZ(1:2:end,:);  %This creates a variable of odd
steptimes.
    Even = SteptimeZ(2:2:end,:);  %This creates a variable of even
steptimes.
    SizeO = size (Odd,1); %This provides the number of rows in the
Odd steptime variable.
    SizeE = size (Even,1); %This provides the number of rows in the
Even steptime variable.

    %The "if elseif" statement below says: if the size (i.e., # of
rows) of
    %the Odd and Even variables are the same, then Odd = Odd (i.e.,
do
    %nothing). If the size of Odd is greater than Even (which will
occur
    %when you have an odd number of rows) then the last row in the
Odd variable is to be removed (Odd(SizeO(1),:) = [];).

    if SizeO == SizeE
        Odd = Odd;
    elseif SizeO > SizeE;
    Odd(SizeO(1),:) = [];
    end

    StridetimeZ = Odd + Even;  %This statement adds each row of the
Odd and Even variables together to provide the series of
    %stride times.
```

```
    AsymZ = (abs(mean(Odd) - mean(Even))/((mean(Odd) +
mean(Even))/2))*100; %This command finds the asymmetry between
    %the mean of the right and left steps.
    avgStepZ = mean(SteptimeZ); %This variable finds the mean of
the series of step times.
    avgStrideZ = mean(StridetimeZ); %This variable find the mean of
the series of stride times.
    COVStepZ = (std(SteptimeZ)/avgStepZ) *100; %This variable finds
the coefficient of variation of the step time series.
    COVStrideZ = (std(StridetimeZ)/avgStrideZ) *100; %This variable
finds the coefficient of variation of the stride time
    %series.
    [CadenceZ] = 1/((size(Negz)/size(SteptimeZ))/60)*60; %This
variable determines the overall cadence of the series
    %based on the total number of samples (in Negz) and the total
number of
    %steps (SteptimeZ).
    StepcountZ = SizeE + SizeO;
    save StepZ.txt SteptimeZ -ascii %This saves the variable
Steptime (containing the series of step times) to a .txt file.
    save StrideZ.txt StridetimeZ -ascii %This saves the variable
Stridetime (containing the series of stride times) to
    %a .txt file.
    %The following line saves the output variables to a ascii .txt
file
    %(figure out how to include a column of labels).
    save OutputZ.txt StepcountZ avgStepZ COVStepZ CadenceZ AsymZ -
ascii
```

STV_ZW CODE (Withers)

```
  %This is the PREFERRED routine for processing the vertical
signal.

    %This script finds the time between the LP filtered Z-axis (AP)
peaks (i.e, the individual Step times) and removes outliers that
are
    %3 SD above and below the median Step time.

    %findpeaks is the function that finds the peaks of the signal.
    %pks = the magnitude of the peaks.
    %locs = the location (i.e, the sample number) of the peaks
(i.e., the
    %peak locations).

    Negz = filtz * -1; %This command flips the LP filtered Z-axis
signal (so that the peak minimums are now maximums).
```

```
    [pks, locs] = findpeaks(Negz,'MinPeakDistance',20,
'MinPeakProminence', 0.25); %This command finds the peaks and
creates variables
    %for the magnitude (pks) and locations (locs) of the peaks. The
command also specifies that there must
    %be a minimum horizontal distance between each peak (i.e,. 20
samples = 0.33 s)
    %and that the peaks must be 0.3 g higher than the lowest value.
    SteptimeZ = diff(locs) * 1/Fs; %This command finds the
differences between the peak locations (i.e., # of samples)
    %and then multiplies this by the sampling rate time. This
provides the
    %series of individual step times.

    ThreshU = median(SteptimeZ) + 3*(std(SteptimeZ)); %This command
finds the median value of the SteptimeZ variable
    %and then adds 3 standard deviations to it.
    OutliersU = find(SteptimeZ > ThreshU); %This creates a variable
that contains the outliers that are greater than the
    %Threshold value.
    SteptimeZ(OutliersU) = [median(SteptimeZ)]; %This command
replaces the outliers in SteptimeZ with the median
    %steptime.
    %The nextseries of commands repeats the above process for
steptimes
    %that are 3 SD's below the median step time.
    ThreshD = median(SteptimeZ) - 3*(std(SteptimeZ));
    OutliersD = find(SteptimeZ < ThreshD);
    SteptimeZ(OutliersD) = [median(SteptimeZ)];

    Odd = SteptimeZ(1:2:end,:);  %This creates a variable of odd
steptimes.
    Even = SteptimeZ(2:2:end,:);  %This creates a variable of even
steptimes.
    SizeO = size (Odd,1); %This provides the number of rows in the
Odd steptime variable.
    SizeE = size (Even,1); %This provides the number of rows in the
Even steptime variable.

    %The "if elseif" statement below says: if the size (i.e., # of
rows) of
    %the Odd and Even variables are the same, then Odd = Odd (i.e.,
do
    %nothing). If the size of Odd is greater than Even (which will
occur
    %when you have an odd number of rows) then the last row in the
Odd variable is to be removed (Odd(SizeO(1),:) = [];).
```

```
    if SizeO == SizeE
        Odd = Odd;
    elseif SizeO > SizeE;
    Odd(SizeO(1),:) = [];
    end

    StridetimeZ = Odd + Even;  %This statement adds each row of the
Odd and Even variables together to provide the series of
    %stride times.

    AsymZ = (abs(mean(Odd) - mean(Even))/((mean(Odd) +
mean(Even))/2))*100; %This command finds the asymmetry between
    %the mean of the right and left steps.
    avgStepZ = mean(SteptimeZ); %This variable finds the mean of
the series of step times.
    avgStrideZ = mean(StridetimeZ); %This variable find the mean of
the series of stride times.
    COVStepZ = (std(SteptimeZ)/avgStepZ) *100; %This variable finds
the coefficient of variation of the step time series.
    COVStrideZ = (std(StridetimeZ)/avgStrideZ) *100; %This variable
finds the coefficient of variation of the stride time
    %series.
    [CadenceZ] = 1/((size(Negz)/size(SteptimeZ))/60)*60; %This
variable determines the overall cadence of the series
    %based on the total number of samples (in Negz) and the total
number of
    %steps (SteptimeZ).
    StepcountZ = SizeE + SizeO;
    save StepZ.txt SteptimeZ -ascii %This saves the variable
Steptime (containing the series of step times) to a .txt file.
    save StrideZ.txt StridetimeZ -ascii %This saves the variable
Stridetime (containing the series of stride times) to
    %a .txt file.
    %The following line saves the output variables to a ascii .txt
file
    %(figure out how to include a column of labels).
    save OutputZ.txt StepcountZ avgStepZ COVStepZ CadenceZ AsymZ -
ascii
```
